# Developing an animal model of Dupuytren’s disease by orthotopic transplantation of human fibroblasts into athymic rat

**DOI:** 10.1186/s12891-015-0597-z

**Published:** 2015-06-07

**Authors:** Latha Satish, Bradley Palmer, Fang Liu, Loukia Papatheodorou, Lora Rigatti, Mark E. Baratz, Sandeep Kathju

**Affiliations:** Department of Plastic Surgery, University of Pittsburgh, 3550 Terrace Street, Scaife Hall, Room no. 685.2, Pittsburgh, PA 15261 USA; Department of Orthopedic Surgery, Allegheny Health Network, Pittsburgh, PA USA; Division of Laboratory Animal Resources, University of Pittsburgh, Pittsburgh, PA USA; Department of Orthopedic Surgery, University of Pittsburgh, Pittsburgh, PA USA; McGowan Institute for Regenerative Medicine, Pittsburgh, PA USA

**Keywords:** Dupuytren’s contracture, Palmar fascia fibrosis, Carpal tunnel syndrome, Fibroblasts, Collagen, Alpha-SMA

## Abstract

**Background:**

Dupuytren’s disease (DD) is a slow, progressive fibroproliferative disorder affecting the palms of the hands. The disease is characterized by the formation of collagen rich- cords which gradually shorten by the action of myofibroblasts resulting in finger contractures. It is a disease that is confined to humans, and a major limiting factor in investigating this disorder has been the lack of a faithful animal model that can recapitulate its distinct biology. The aim of this study was to develop such a model by determining if Dupuytren’s disease (DD)- and control carpal tunnel (CT)-derived fibroblasts could survive in the forepaw of the nude rats and continue to exhibit the distinct characteristics they display in *in vitro* cultures.

**Methods:**

1x10^7^ fluorescently labeled DD- and CT-derived fibroblasts were transplanted into the left and right forepaws of nude rats respectively. Cells were tracked at regular intervals for a period of two months by quantifying emitted fluorescent signal using an IVIS imaging system. After a period of 62 days rat forepaw connective tissues were harvested for histology and total RNA was isolated. Human-specific probes were used to perform real time RT-PCR assays to examine the expression patterns of gene products associated with fibrosis in DD. Rat forepaw skin was also harvested to serve as an internal control.

**Results:**

Both CT- and DD-derived fibroblasts survived for a period of 62 days, but DD-derived cells showed a significantly greater level of persistent fluorescent signal at the end of this time than did CT-derived cells. mRNA expression levels of α-smooth muscle actin (α-SMA), type I- and type III- collagens were all significantly elevated in the forepaw receiving DD cord-derived fibroblasts in comparison to CT-derived fibroblasts. Masson’s trichrome stain confirmed increased collagen deposition in the forepaw that was injected with DD cord-derived fibroblasts.

**Conclusions:**

For the first time we describe an animal model for Dupuytren’s disease at the orthotopic anatomical location. We further show that gene expression differences between control (CT) and diseased (DD) derived fibroblasts persist when these cells are transplanted to the forepaw of the nude rat. These preliminary findings indicate that, with further refinements, this animal model holds promise as a baseline for investigating novel therapeutic regimens to determine an effective strategy in treating DD.

## Background

Dupuytren’s disease (DD) is a complex, progressive, fibroproliferative disorder which commonly affects populations of northern European descent and has a prevalence of >7 % in the United States [1]. DD affects the palmar fascia of the hand resulting in digital flexion contractures. DD is considered a benign, heritable fibrosis [2] inherited in an autosomal dominant fashion with variable penetrance [3].

Dupuytren’s disease generally begins as a hard nodule on the palm and slowly progresses into a fibrous cord that pulls the affected digit into a flexed contracted position [4, 5]. The digits most commonly affected are the ring and small fingers, and contractures usually occur at either the metacarpophalangeal (MP) joint, proximal interphalangeal (PIP) joint, or rarely the distal interphalangeal joint. The nodule is a relatively vascular tissue containing a dense population of fibroblasts, with a high proportion being myofibroblasts [6, 7], specialized cells that express α-smooth muscle actin (α-SMA) [8] and are the likely effectors of the tissue contraction characteristic of DD. The nodule may develop into a collagen-rich fibrotic cord as the disease progresses over time, with a persistent abundance of myofibroblasts. Recent studies show that cells derived from primary nodule and cord have similar gene expression profiles [9], indicating that both nodule- and cord-derived fibroblasts are appropriate subjects of study in examining the progression of DD.

The exact etiopathogenesis of DD remains unknown. In addition to a genetic predisposition, environmental and behavioral factors (eg. alcohol consumption and manual labor) [10, 11] have also been implicated in DD, as have certain co-morbid conditions (eg. diabetes, epilepsy and hypercholesterolemia) [12–14]. At a more basic level, DD is marked by a disturbance in collagen metabolism, and by changes in other extracellular matrix protein levels, including fibronectin [15] and periostin [16]. Dolmans et al. (2011) [17] identified nine different loci involved in genetic susceptibility to DD of which six are in the vicinity of genes encoding proteins in the Wnt-signaling pathway, suggesting that malfunctioning of this pathway may be a key factor for the progression and/or recurrence of DD. But the most important factor linked to progression of DD remains the proliferation of fibroblasts and their transformation to a myofibroblast phenotype, as evidenced by increased levels of α-SMA [18]. Our previous data examining the global gene expression patterns of DD-derived fibroblasts versus control carpal tunnel (CT)-derived fibroblasts confirms that there are numerous differences in their transcriptomes even in *in vitro* culture, indicating a stable intrinsically distinct disease physiology for DD-cells [19, 20].

Currently there is no treatment available that can successfully cure, prevent progression or recurrence of DD. In mild cases treatment is only observation, but as the disease progresses needle fasciotomy or open surgery becomes necessary to release contracture caused by the fibrosis. Multiple surgical techniques have been described for the treatment of DD that focus on resecting the fibrotic tissue with the goal of improving finger extension, but none have proven to be consistently more effective than others [21]. Recently, direct injection of Clostridium histolyticum collagenase (CHC) capable of lysing DD cords has been used with some promise, although long term follow-up of these patients reveals some significant complications that may necessitate careful evaluation [22–24]. Alternative treatment options, including nonsurgical molecular therapies to halt the progression and recurrence of DD, would be a welcome advance.

The development of such therapies has been slowed in part by the lack of a well-described animal model in which they may be initially assessed, as DD is a condition unique to humans, without counterpart in the animal kingdom. We therefore investigated the characteristics of human DD fibroblasts transplanted into an immunodeficient animal host to see if they could maintain their distinct disease phenotype compared to control cells harvested from fascia from patients undergoing carpal tunnel release. We monitored their persistence over time and examined their molecular and histologic profiles at the end of some two months after their orthotopic transplantation into nude rat forepaw.

## Methods

### Clinical specimens and primary cell culture

Dupuytren’s disease (DD) cord and carpal tunnel (CT) fascial tissue samples were surgically resected at the Division of Upper Extremity Surgery, Department of Orthopaedic Surgery, Allegheny General Hospital, Pittsburgh, PA. All subjects provided written informed consent and specimens were collected with the approval of the Allegheny-Singer Research Institute’s Institutional Review Board (IRB protocol RC-4040) involving human subjects. The study protocol conformed to the ethical guidelines of the1975 Declaration of Helsinki.

Primary cultures of fibroblasts were isolated from freshly resected DD cord tissue and normal palmar fascia (CT cells) as previously described [25]. The cultures were maintained in α-MEM medium supplemented with 10 % fetal bovine serum (FBS, Invitrogen™, Life Technologies, Grand Island, NY) and 1 % antibiotic-antimycotic solution (Sigma-Aldrich, St Louis, MO). Cultures were maintained until a maximum of six passages, during which no change in the cell morphology was observed. Cells between passages 2–5 were used for *in vitro* and transplantation experiments. While in culture, fibroblasts were tagged *in vitro* with fluorescent Lipophilic Cell Tracer Vybrant™ DiR (D12731; Invitrogen™, Life Technologies) as per the manufacturer’s instructions. DiR is a dialkylcarbocyanine dye with excitation and emission maxima in the near infrared fluorescence region and this high wavelength prevents conflation with any autofluorescence of tissue.

### The CellTiter 96® non-radioactive cell proliferation assay

Cell proliferation/viability was examined using the CellTiter 96® Non-Radioactive Cell Proliferation Assay kit from Promega Corporation (Madison, WI). This assay is based on the cellular conversion of a tetrazolium salt into a formazan product that is easily detected using a 96-well plate reader. Cells were plated in 24-well plates at a density of 5x10^4^ cells/well, grown for 24 h, and then treated with DiR (10 μg/ml) for 30 min and/or for 24 h. After the exposure periods, the cells were photographed and replaced with fresh medium to perform the assay. Dye solution (20 μl) was added and was incubated at 37 °C for an additional 2 h. As a final step 133 μl solubilization/stop solution was added and incubated at 37 °C for 1.5 h. Results were obtained by measuring absorption at 570 nm by aliquoting 100 μl into a Corning 96 well flat transparent plate.

### Nude rats

All studies on “nude” (rnu/rnu athymic) rats were performed with approval by the Institutional Animal Care and Use Committee (IACUC) of the Allegheny General Hospital. The outbred nude rats were commercially purchased from Charles River Laboratories (Wilmington, MA). All animals were male, weighed between 250 and 350 g, and were acclimatized for 2 weeks prior to experiments. Animals were housed in pathogen-free individual cages.

### Animal experiments

Male athymic rats (n = 6) were anesthetized with an intraperitoneal injection containing a loading dose of ketamine at 50 mg/kg and xylazine at 5 mg/kg. Fibroblasts (1x10^7^) derived from both DD-cord and CT tissues labeled with DiR were transplanted subcutaneously using a tuberculin syringe into the forepaws of the nude rats. In each animal, the right forepaw was implanted with CT-derived fibroblasts and the left forepaw received DD-cord derived fibroblasts. All animals underwent IVIS imaging of their forepaws at regular intervals by placing the animal in a light-tight chamber, and images were generated over a 10 s exposure using a cryogenically cooled charge-coupled camera (IVIS Lumina II, Caliper Life Sciences, Hopkinton, MA) to quantify photons emitted by the animal. Animals were anesthetized with inhaled 2 % isoflurane for imaging using a nose cone delivery device. The IVIS camera was maintained at standard settings, as follows: imaging mode fluorescent, exposure time 10 s, and binning high with field of view high. The visual output represents the number of photons emitted/cm^2^ as a pseudocolor image where the maximum is red and minimum is purple. Once compiled, images were quantified for detection of fluorescent signal emitted from the forepaw using Xenogen Living Image® software.

At 62 days post transplantation, animals were sacrificed and the left and right rat forepaw tissues were collected separately for further histological and real time RT-PCR analyses. In harvesting the specimens with 3.5x loupe magnification, the overlying rat skin was dissected away from subjacent structures and retained as a control. The remaining forepaw tissues, from the carpus to the base of the digits, were harvested en bloc up including immediately subcutaneous tissues down to bone so as to minimize variability in tissue sampling. The collected tissues from all the six animals were divided for histology and real time RT-PCR analysis.

### Histology

Rat forepaw tissues from both DD- and CT-injected animals, as well as control uninjected animals, were harvested at 62 days post transplantation and fixed in 10 % neutral buffered formalin for histological evaluation. Tissue samples embedded in paraffin were sectioned at a thickness of 5 μm and the sections were simultaneously stained with Masson’s trichrome to evaluate collagen deposition following standard protocols.

### RNA extraction

Tissues harvested from the rat forepaws were stored immediately in RNAlater® (Ambion, Austin, TX). Total RNA was extracted using the RNeasy Mini Kit (Qiagen Inc., Valencia, CA) following manufacturer’s protocol after homogenization using a homogenizer. The quality and quantity of total RNA obtained were determined by measuring the OD 260/OD 280 ratio using an ND-1000 spectrophotometer (Nanodrop Technologies Inc., Wilmington, DE) and by capillary electrophoresis with the Agilent 2100 BioAnalyzer (Agilent Technologies Inc., Palo Alto, CA) and a Nano6000 RNA chip, with all sample RNA values >7.0.

### Quantitative real time RT-PCR (qRT-PCR)

qRT-PCR was performed using kits obtained from Applied Biosystems® Life Technologies (Grand Island, NY) utilizing FAM™Taqman®MGB probes and a Taqman® Universal PCR master Mix. Probes were used for three gene products, namely human Type I collagen, human Type III collagen and human α-smooth muscle actin (α-SMA). Human GAPDH was used as the endogenous normalization control for these studies. Reverse transcription was performed using 100 ng/μl of total RNA and with M-MLV-reverse transcriptase (Invitrogen™ Life Technologies). Subsequent PCR amplification and detection of template was carried out using Applied Biosystems transcript-specific assays including: type I collagen (COL1A2) assay (ID-Hs 01028971_m1), type III collagen (COL3A1) assay (ID-Hs 00947393_g1) and α-SMA assay (ID-Hs 00426835_g1) using 15 ng/μl of cDNA and 20X final concentration of Gene Expression Mix which contains both forward and reverse primers along with the gene-specific probes adjusted to a final volume of 15.0 μl. The reaction set up and the thermal cycling protocol was as previously described [19, 20]. Using the comparative critical cycle (Ct) and by relative quantification methods (RQ), the expression levels of the target genes were normalized to the GAPDH endogenous control (ID-HS99999905_m1) and the relative abundance was calculated. Data were analyzed using the 7900 HTSDS software version 2.1 provided by Applied Biosystems.

### Statistical analyses

Statistical analyses were performed using Student’s *t* test function using Microsoft Excel program for qRT-PCR analyses. p < 0.05 was considered statistically significant. Repeated measures one-way ANOVA was used to determine the statistical significance for the data collected using IVIS imaging. This was accomplished utilizing GraphPad Prism 6.

## Results

### Labelling of DD and CT cells with DiR dye had no effect on cell viability or proliferation

**Fig. 1:**
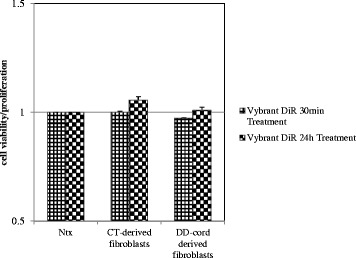
Lipophilic cell tracer DiR® does not affect fibroblast proliferative capacity. Cell viability and proliferative capacity of Vybrant DiR® labeled and unlabeled CT- and DD-derived fibroblasts was compared *in vitro*. No significant difference was observed in viability or proliferative capacity between the labeled and unlabeled cell populations for both CT- and DD-cells at either 30 min or 24 h. Studies were performed on four independent cultures obtained from each of the cell types; values are mean ± SEM of two separate experiments performed in triplicate

In order to track our populations of cells over time *in situ* in the forepaw, we first labelled them with Vybrant™ DiR, a lipophilic dye that is only weakly fluorescent in aqueous solution but is strongly fluorescent and photostable when present in cell membranes. DiR uniformly and effectively labeled both DD and CT cells, with no light detected in the unlabeled cell population and with no apparent distortion in cell morphology [26]. We further confirmed that DiR did not have any significant effect on the proliferative capacity of either DD or CT cells, nor did it elicit any cytotoxic effect when compared to unlabeled cell populations (Fig. 1).

### DD- and CT-derived fibroblasts successfully persisted in the forepaw of nude rats

**Fig. 2:**
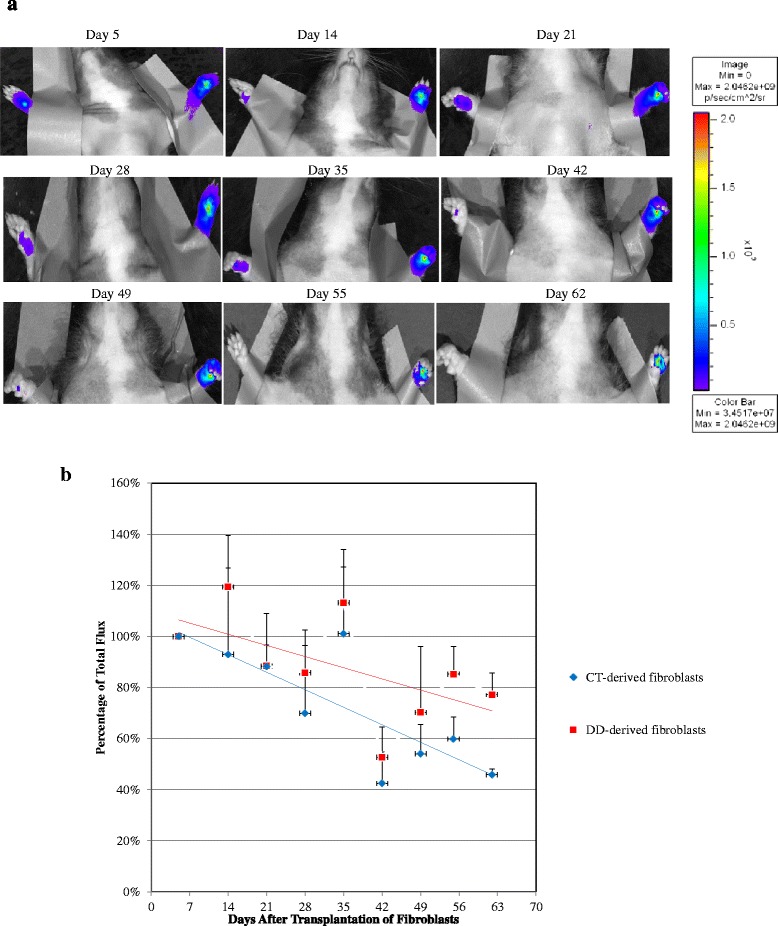
Imaging of human fibroblasts transplanted to nude rat forepaws shows cellular persistence for 62 days. Non-invasive *in vivo* imaging of the forepaws receiving DiR labeled CT-and DD-derived fibroblasts showed persistence of both populations of cells to 62 days (n = 6). The emitted fluorescent signal gradually declined over time for both cell types, but at day 62 DD-cells showed a significantly greater presence than CT-cells. (**a**) A representative image of the emitted fluorescent signals over time. Animals were injected with DD-derived fibroblasts in the left forepaw and with CT-derived fibroblasts on the right forepaw. (**b**) Quantitative assessment of emitted fluorescent signal over time, with day 5 set at 100 % of total flux. Statistical analyses using ANOVA showed a significant difference in the intensity of persisting fluorescence signal over time between the forepaw that received CT- and DD-derived fibroblasts. p value was < 0.0001

We had previously demonstrated that administration of both 1.0x10^7^ and 1.5x10^7^ DD-derived fibroblasts in nude rat forepaws yielded successful persistence of cells over ~8 weeks as evidenced by continued detection of fluorescent signal emitted from the forepaw [26]. In this study we investigated differences in the survival ability between control (CT-derived fibroblasts) and diseased (DD-derived fibroblasts) samples. CT- and DD -derived fibroblasts were injected into the right and left forepaws of nude rats respectively which were then imaged at regular intervals. Both CT- and DD-derived cells survived in the forepaws over almost 9 weeks (Fig. 2a). Quantitative assessment of emitted fluorescent signal over time was calculated, with day 5 set at 100 % of total flux. The amount of fluorescent signal did show some decline over time in both populations, but DD-derived cells showed significantly less decline (therefore greater persistence) at day 62, retaining almost 80 % of their original signal intensity compared to less than 50 % for CT-derived cells (Fig. 2b). There was no visual evidence of any significant migration of the cell populations outside the forepaw zone.

### Masson’s trichrome staining showed increased collagen deposition in the forepaw that received DD- derived fibroblasts

**Fig. 3:**
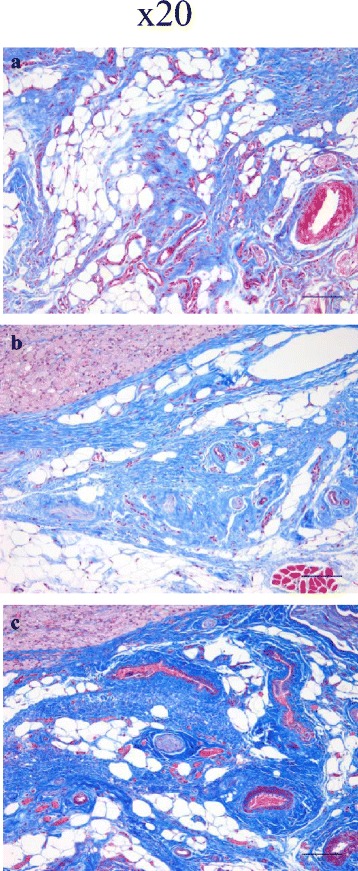
Histologic evaluation of fascial tissues injected with DD-derived fibroblasts shows increased collagen deposition. A representative histochemical Masson’s trichrome stain of forepaw tissues harvested from three animals obtained from (**a**) untreated forepaw (**b**) forepaw implanted with CT-derived fibroblasts and (**c**) forepaw implanted with DD- derived fibroblasts is shown in x20 magnification. Tissues harboring DD-cells showed markedly darker blue staining, signifying increased collagen deposition

One of the parameters used to determine the degree of fibrosis is the amount of collagen deposited. Tissue specimens obtained from forepaw injected with CT- and DD-derived fibroblasts as well as untreated fascial control tissues were stained simultaneously with Masson’s trichrome to determine the pattern of collagen deposition; representative images are shown in Fig. 3. The connective tissue within the palmar fascia of the DD-injected forepaw showed consistently and appreciably darker staining (Fig. 3c) when compared to the forepaw that received CT-derived fibroblasts (Fig. 3b) and the control forepaw (Fig. 3a); no consistent difference was appreciated between CT-transplanted forepaws and native control fascia. This clearly indicates a denser deposition of connective tissue (mainly collagen) within the palmar fascia of the DD-derived fibroblasts, an indication that greater fibrosis is occurring.

### DD-derived fibroblasts showed persistently elevated expression of multiple genes involved in fibrosis and contraction after transfer to the forepaw

We next sought to determine if the differential physiology between CT- and DD-derived fibroblasts seen in *in vitro* culture, presumably reflective of their distinct fibroproliferative capacities, would persist after transplantation into the nude rat forepaw. We therefore used qRT-PCR to examine the expression profiles of three genes known to be significantly over-expressed in DD physiology, specifically the Type I and Type III collagens, as well as α-SMA. We compared CT-injected forepaws to DD-injected forepaws, and also compared the patterns of expression of these genes after 62 days in forepaws to their expression patterns in baseline cultured CT- and DD-fibroblasts. We also examined the overlying rat forepaw skin (in which no human cellular material would be expected; although rat fascia may be a more ideal control substrate, skin is also known to express collagen and α-SMA in abundance).

**Fig. 4:**
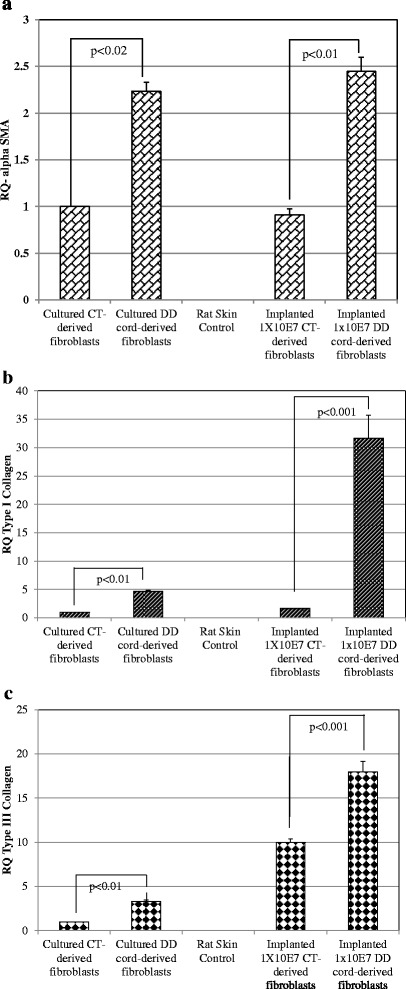
Quantitative RT-PCR of transplanted forepaw tissues shows persistently increased expression of fibrosis genes in DD-cells versus CT-cells. Real time qRT-PCR was performed to measure the mRNA expression levels of α-SMA (**a**), type I collagen (**b**) and type III collagen (**c**), all of which were markedly elevated in DD-cells compared to CT-cells. For quantitative analysis of gene expression, the comparative threshold cycle (Ct) method for relative quantification was used. The expression of the target genes was normalized to GAPDH expression. Values represent the mean ± SEM of two separate experiments performed on RNA derived from the forepaw tissues of four animals, each performed in triplicate. Parallel *in vitro* cultures of the CT- and DD-derived fibroblasts transplanted *in vivo* were used as controls. P value less than 0.05 was considered as statistically significant

Quantitative real-time RT-PCR expression analyses of α-SMA, type I and type III collagen are shown in Fig. 4. No expression at all was detected for any of the three genes in rat skin, confirming the human-specificity of the assays. Cultured DD-cells showed markedly higher levels of all three genes than cultured CT-cells, as expected. These patterns generally persisted after transplantation into the forepaw, and were amplified in some instances. DD-cells in the forepaw continued to express over twice as much α-SMA as CT-cells (Fig. 4a). DD-cells in the forepaw similarly expressed much more Type I collagen than CT-cells, and indeed showed an apparent increase over even their baseline expression when maintained in culture (Fig. 4b). Both DD- and CT-cells showed an apparent increase in Type III collagen expression than their baseline levels in culture, but here again DD-cells in the forepaw showed significantly higher levels than CT-cells in the forepaw (Fig. 4c). In total, DD-cells, after 62 days in the forepaw, continued to demonstrate significantly increased expression of each of these markers of fibrosis than similarly transplanted CT-cells.

## Discussion

A major factor hindering the development of novel therapies for DD is the lack of a well-established *in vivo* animal model to study the progression and recurrence of the disease. Although there have been multiple previous attempts to develop such a model, these have all been marked with certain features that have limited their utility. As a first attempt Larsen et al. (1960) [27] reported Dupuytren’s-like lesions in monkeys after traumatic disruption of palmar fascial fibers. Although this model was situated in the appropriate anatomical location, there is no underlying Dupuytren’s cell type involved, and this model may be better regarded as one of post-traumatic fibrosis rather than of a distinct fibroproliferative disorder such as Dupuytren’s.

Subsequent models have used explanted human Dupuytren’s tissue as the basis for their pathology, but have introduced them into heterotopic locations in the animal hosts. Kischer et al. (1989) [28] determined that DD tissues implanted into subcutaneous pockets in the suprascapular area of nude mice maintained their histologic character and electron microscopic structure. Although the size of implants reduced in size over time, the appearance of fibroblasts and the spatial pattern of collagen remain unchanged. The authors concluded that the use of implants into nude mice maybe useful for further experimental studies of DD but to date there have been no follow up studies with this model. Kuhn et al. (2001) [29] used a vascularized sandwich flap model in the abdominal skin of nude rats to maintain viable DD-affected palmar fascia tissue explants for up to 60 days. The authors also demonstrated that perfusion of the explanted DD tissue by TGFβ_2_ upregulated collagen I and III, and that perfusion with antibody against TGFβ_2_ prevented the same increase. Both of these studies used immunodeficient hosts in a manner similar to this investigation, but used whole tissues rather than focusing on a specific cell type (fibroblasts/myofibroblasts) and placed them into an aphysiological milieu rather than the orthotopic location of the palmar fascia.

Recently, Karkampouna et al. (2014) [30] have reported an *ex vivo* “clinical trial” system by culturing DD specimens after surgical removal, observing that the tissue so cultured preserved the pathological status of the disease by maintaining the complex organization of the ECM and its three-dimensional (3D) structure. The authors also demonstrated that targeting TGFβ_1_ receptor expression in cultured DD specimens by antisense oligonucleotide-mediated exon skipping showed a specific reduction in fibrotic proteins and caused a reversal of the fibrotic phenotype. This is an encouraging result, but their system extends for only seven days in culture with treatments administered over three days time. It remains to be seen whether it can be adapted to the longer time periods over which DD manifests, and it may not fully capture all factors in play when evaluating *in vivo* disease progression and recurrence.

In our effort to develop an animal model of DD we have previously utilized athymic “nude” rats and have showed that DD-derived fibroblasts can persist in the forepaw of the animals for up to 8 weeks, retaining their myofibroblast phenotype [26]. In the present study, we demonstrate that DD cells show greater persistence over time than control CT cells, and retain a distinctly pro-fibrotic physiology. While we did see molecular and histologic evidence of building fibrosis with DD cells, we did not see the development of a frank tissue contracture analogous to the clinical presentation in humans. This may be because our model incompletely mimics the human pathophysiology, or it may be that more time is required for that degree of fibrosis and contracture to manifest. Dupuytren’s is after all a disease with a slow and progressive onset, often taking years to become clinically significant, and for practical reasons our studies here only extend to nine weeks, a relatively lengthy experimental period but still short compared to the clinical condition. Ongoing studies to allow these cells to persist *in situ* for even longer periods of time may help to clarify these possibilities. Because even DD cells do show some element of decline over time, it may even become necessary to administer repeated doses of DD cells to achieve frank tissue contracture.

But in some senses this may not be necessary. The model we describe here for the first time places the principal effector cells of DD in the physiologically relevant orthotopic anatomical location, and follows their behavior over time. The increased evidence of fibrosis we see with DD cells compared to CT cells suggests that a meaningful recapitulation of DD physiology is occurring, and can already serve as a baseline against which novel therapeutic interventions can be tested. Any agent found to diminish the DD-dependent fibrotic characteristics seen thus far may be regarded as a potential candidate for clinical translation. An advantage of this model in that regard is that any therapy to be examined must first be made accessible to the DD cells in the subcutaneous/palmar fascial tissues, overcoming the skin barrier even as it would have to be in an actual clinical application. This might be accomplished by subcutaneous injection (as the cells themselves were injected), or agents manufactured to penetrate the skin barrier could be evaluated after topical administration. We hope that further refinements of this model and its use in testing new molecular therapies can help to accelerate improvements in the care of patients with Dupuytren’s disease.

## Conclusions

For the first time this study describes an animal model for Dupuytren’s disease at the orthotopic anatomical location. We also show that gene expression differences between control (CT-derived) and diseased (DD-derived) fibroblasts persist when these cells are transplanted to the forepaw of the nude rat. These preliminary findings indicate that, with further refinements, this animal model holds promise as a baseline for investigating novel therapeutic regimens to determine an effective strategy in treating DD.
